# Analysis of Hospital-Level Readmission Rates and Variation in Adverse Events Among Patients With Pneumonia in the United States

**DOI:** 10.1001/jamanetworkopen.2022.14586

**Published:** 2022-05-31

**Authors:** Yun Wang, Noel Eldridge, Mark L. Metersky, David Rodrick, Constance Faniel, Sheila Eckenrode, Jasie Mathew, Deron H. Galusha, Anila Tasimi, Shih-Yieh Ho, Lisa Jaser, Andrea Peterson, Sharon-Lise T. Normand, Harlan M. Krumholz

**Affiliations:** 1 Center for Outcomes Research and Evaluation, Yale New Haven Hospital, New Haven, Connecticut; 2 Section of Cardiovascular Medicine, Department of Internal Medicine, Yale School of Medicine; New Haven, Connecticut; 3 Richard and Susan Smith Center for Outcomes Research in Cardiology, Division of Cardiology, Beth Israel Deaconess Medical Center, Harvard Medical School, Boston, Massachusetts; 4 Department of Biostatistics, Harvard T.H. Chan School of Public Health, Boston, Massachusetts; 5 Agency for Healthcare Research and Quality, Department of Health and Human Services, Washington, DC; 6 now with Defense Health Agency, Falls Church, Virginia; 7 Division of Pulmonary and Critical Care Medicine, University of Connecticut School of Medicine, Farmington; 8 Centers for Medicare & Medicaid Services, Department of Health and Human Services, Baltimore, Maryland; 9 now with Health Resources and Services Administration Department of Health and Human Services, Washington, DC; 10 Corporate Business Services, Yale–New Haven Health System, New Haven, Connecticut; 11 Department of Pharmacy, Griffin Hospital, Derby, Connecticut; 12 Hartford Healthcare, Trumbull, Connecticut; 13 St Vincent’s Hospital, Bridgeport, Connecticut; 14 Department of Health Care Policy, Harvard Medical School, Boston, Massachusetts; 15 Department of Health Policy and Management, Yale School of Public Health, New Haven, Connecticut

## Abstract

**Importance:**

It is known that hospitalized patients who experience adverse events are at greater risk of readmission; however, it is unknown whether patients admitted to hospitals with higher risk-standardized readmission rates had a higher risk of in-hospital adverse events.

**Objective:**

To evaluate whether patients with pneumonia admitted to hospitals with higher risk-standardized readmission rates had a higher risk of adverse events.

**Design, Setting, and Participants:**

This cross-sectional study linked patient-level adverse events data from the Medicare Patient Safety Monitoring System (MPSMS), a randomly selected medical record abstracted database, to the hospital-level pneumonia-specific all-cause readmissions data from the Centers for Medicare & Medicaid Services. Patients with pneumonia discharged from July 1, 2010, through December 31, 2019, in the MPSMS data were included. Hospital performance on readmissions was determined by the risk-standardized 30-day all-cause readmission rate. Mixed-effects models were used to examine the association between adverse events and hospital performance on readmissions, adjusted for patient and hospital characteristics. Analysis was completed from October 2019 through July 2020 for data from 2010 to 2017 and from March through April 2022 for data from 2018 to 2019.

**Exposures:**

Patients hospitalized for pneumonia.

**Main Outcomes and Measures:**

Adverse events were measured by the rate of occurrence of hospital-acquired events and the number of events per 1000 discharges.

**Results:**

The sample included 46 047 patients with pneumonia, with a median (IQR) age of 71 (58-82) years, with 23 943 (52.0%) women, 5305 (11.5%) Black individuals, 37 763 (82.0%) White individuals, and 2979 (6.5%) individuals identifying as another race, across 2590 hospitals. The median hospital-specific risk-standardized readmission rate was 17.0% (95% CI, 16.3%-17.7%), the occurrence rate of adverse events was 2.6% (95% CI, 2.54%-2.65%), and the number of adverse events per 1000 discharges was 157.3 (95% CI, 152.3-162.5). An increase by 1 IQR in the readmission rate was associated with a relative 13% higher patient risk of adverse events (adjusted odds ratio, 1.13; 95% CI, 1.08-1.17) and 5.0 (95% CI, 2.8-7.2) more adverse events per 1000 discharges at the patient and hospital levels, respectively.

**Conclusions and Relevance:**

Patients with pneumonia admitted to hospitals with high all-cause readmission rates were more likely to develop adverse events during the index hospitalization. This finding strengthens the evidence that readmission rates reflect the quality of hospital care for pneumonia.

## Key Points

**Question:**

Do patients with pneumonia who are admitted to hospitals with higher risk-standardized readmission rates have higher rates of adverse events?

**Findings:**

This cross-sectional study of 46 047 patients found that a 1-IQR increase in a hospital’s readmission rate was associated with a 13% higher relative risk of adverse events for patients and 5 more adverse events per 1000 discharges.

**Meaning:**

In this study, patients with pneumonia admitted to hospitals with a high all-cause readmission rate were more likely to develop adverse events during the index hospitalization.

## Teaser

This cross-sectional study evaluates whether patients with pneumonia admitted to hospitals with higher risk-standardized readmission rates had a higher risk of adverse events.

## Introduction

The Centers for Medicare & Medicaid Services (CMS) have publicly reported hospital risk-standardized readmission rates since 2008. The measures were then subsequently incorporated into the Hospital Readmission Reduction Program, which levies penalties on those institutions with higher than average rates.^1,2,3,4^ These measures, however, have been controversial, with some experts suggesting that they have inadequate adjustment for readmission risk.^5,6^ Nevertheless, there is evidence that the risk of readmission varies by hospital after accounting for patient admission severity, and there remains a need to determine whether a hospital’s readmission rate is reflective of its overall quality.

One approach to further investigation of the utility of the readmission measure is to determine its association with other relevant performance metrics. For example, it is known that hospitalized patients who experience safety-related adverse events are at greater risk of readmission.^7,8,9,10^ What is not known is whether the CMS readmission measure conveys information about a hospital’s safety. Specifically, do patients admitted to hospitals with a higher risk-standardized 30-day all-cause readmission rate have a higher risk of a safety event while hospitalized? Such an association would help to strengthen the utility of the readmission measure and identify a potential target for some hospitals to improve their readmission rates. This approach would be important, as most readmission reduction programs focus on interventions at the time of discharge, rather than during hospitalization.

Accordingly, we used data from the Medicare Patient Safety Monitoring System (MPSMS) and data from the CMS to investigate the association between hospital performance on risk-standardized 30-day all-cause readmission rates and patient adverse events for patients hospitalized with pneumonia. We focused on pneumonia for 2 reasons: (1) it is a leading cause of both hospitalization and death in the United States, and (2) it has been included in both MPSMS and CMS data. MPSMS is the nation’s largest patient-safety database and includes hospital medical records of all-payer patients aged 18 years and older. CMS data includes hospital performance on readmissions for Medicare fee-for-service patients across over 4000 Medicare-certified hospitals. Our research question was: did patients with pneumonia admitted to hospitals with higher risk-standardized readmission rates have a higher risk of adverse events during the hospitalization?

## Methods

### Study Sample

The Yale University institutional review board reviewed the study protocol and granted a waiver of informed consent based on the retrospective nature and minimal risk of the study. The study followed the guidelines for cohort studies described in the Strengthening the Reporting of Observational Studies in Epidemiology (STROBE) guidelines.^11^

The MPSMS data, at the patient level, includes 21 common in-hospital adverse event measures (eTable 1 in the Supplement) jointly developed by federal agencies and private health care organizations.^12,13^ MPSMS medical records were obtained from the CMS Hospital Inpatient Quality Reporting program, which includes a multistage random sample of all-payer patients hospitalized for acute myocardial infarction, heart failure, pneumonia, major surgical care, and all other conditions. Each hospital contributed an approximately equal number of randomly selected records to the MPSMS.

The CMS risk-standardized all-cause readmission rates for patients discharged alive with pneumonia, available from the Hospital Compare website,^14^ and at the hospital level, which includes hospital-specific risk-standardized 30-day all-cause readmission rates from acute-care hospitals that treated at least 25 Medicare fee-for-service patients aged 65 years and older. CMS used a 3-year period–combined data set to report readmission rates for each hospital. We used 5 reporting periods: (1) July 1, 2010, to June 30, 2013; (2) July 1, 2012, to June 30, 2015; (3) July 1, 2013, to June 30, 2016; (4) July 1, 2015, to June 30, 2018; and (5), July 1, 2017, to December 31, 2019. Not all hospitals were repeated in each reporting period. To maximize the number of hospitals from both MPSMS and readmissions data, we combined these 5 period data sets to a multiple period data set from July 1, 2010, to December 31, 2019. If a hospital was in more than 1 period, we averaged its readmission rates, weighted by the total discharges in each period. Overall, hospital readmission rates varied from 17.4% in 2010 to 2013 to 16.7% in 2017 to 2019. Approximately 93% of hospitals were repeated 3 or more times in the study.

We limited MPSMS data to patients with a principal discharge diagnosis code of pneumonia^15^ from July 1, 2010, through December 31, 2019, to align with the CMS data. Patients who acquired pneumonia as a complication during hospitalization were not included in the study.

### Patient and Hospital Characteristics

MPSMS patient characteristics include demographics (age, sex, and self-reported race, categorized as Black, White, and other [includes any identified race not included in the aforementioned categories and multiracial]), common clinical comorbidities (heart failure, obesity, coronary artery disease, kidney disease, cerebrovascular disease, chronic obstructive pulmonary disease, cancer, and diabetes), and smoking status. We included the race information in this study because it is a part of demographic characteristics. Hospital characteristics were obtained from the American Hospital Association’s 2010 to 2017 Annual Survey Database and include teaching status (teaching vs nonteaching), Joint Commission certification status, geographic location (urban vs rural), ownership (public, private, vs not-for-profit), beds, and ability to perform coronary artery bypass graft surgery and percutaneous coronary intervention. For 4 hospitals with missing characteristics, we used additional publicly available data sources, including CMS hospital performance data, to obtain their characteristics. Missing information on the number of beds (for 0.03% of hospitals) was imputed using multiple imputation with 10 imputations.

### Outcome and Hospital Performance on Readmissions

Our outcome was hospital-acquired adverse events, defined by 2 indicators: (1) the rate of occurrence of adverse events for which patients were at risk and (2) the number of adverse events per 1000 discharges. The first indicator was at the individual adverse event measurement level, and the second indicator was at the hospital level, allowing us to assess the research question at both patient and hospital levels.

CMS measures hospital-specific 30-day all-cause readmissions based on the risk-standardized method for profiling hospitals (eAppendix 1 in the Supplement).^15,16,17^ We classified each hospital into 1 of 3 mutually exclusive categories by its risk-standardized readmission rate: (1) low, if the rate was less than the 25th percentile of the overall rate; (2) high, if the rate was greater than 75th percentile of the overall rate; and (3) average, if otherwise.

### Statistical Analysis

We performed descriptive analyses to compare patient and hospital characteristics across hospital performance categories described previously. To determine whether hospital performance on readmissions was associated with patients’ risk of adverse events, we fit a mixed-effects model with a logit-link function and random patient and hospital intercepts to model the probability of occurrence of adverse events as a function of hospital readmission rates, adjusting for patient and hospital characteristics. We included a time variable, ranging from 0 (year 2010) to 9 (year 2019) in the model to account for secular trends in adverse events and seasonal indicators (winter, spring, and fall, with summer as the reference) to account for seasonal variation in pneumonia. To determine whether hospital performance on readmissions was associated with hospital performance on adverse events, we regressed the hospital-specific risk-standardized number of adverse events per 1000 discharges (eAppendix 2 in the Supplement) as a function of the hospital-specific risk-standardized readmission rate, adjusting for the hospital characteristics. To address a potential bias resulting from the CMS readmission rates being calculated based solely on Medicare patients aged 65 years or older and patients in MPSMS data being 18 years and older, we conducted a secondary analysis restricting MPSMS data to patients 65 years or older and repeated the previously described analyses.

Because we linked the MPSMS adverse event data with the CMS readmission data at the hospital level, and the readmission data was aggregated as 3-year combined, it is possible that a hospital’s readmission rate was not measured prior to a patient being admitted. To address this consideration, we conducted a sensitivity analysis by including only hospitals in the first CMS reporting period (July 1, 2010, to June 30, 2013) and the MPSMS data after the first reporting period (July 1, 2013, to December 31, 2019). This approach ensures that all patients in the MPSMS data were admitted to hospitals with preexisting information on their performance on readmissions.

Estimated model coefficients were scaled to represent the association between changes in adverse events and 1 IQR change (ie, an increase in readmission rate from the 25th percentile to the 75th percentile) in the hospital risk-standardized readmissions. Analyses were conducted using SAS version 9.4 (SAS Institute). No adjustments have been made for multiplicity of estimation; statistical tests used a 2-sided α of .05.

## Results

### Study Sample

**Figure 1.**
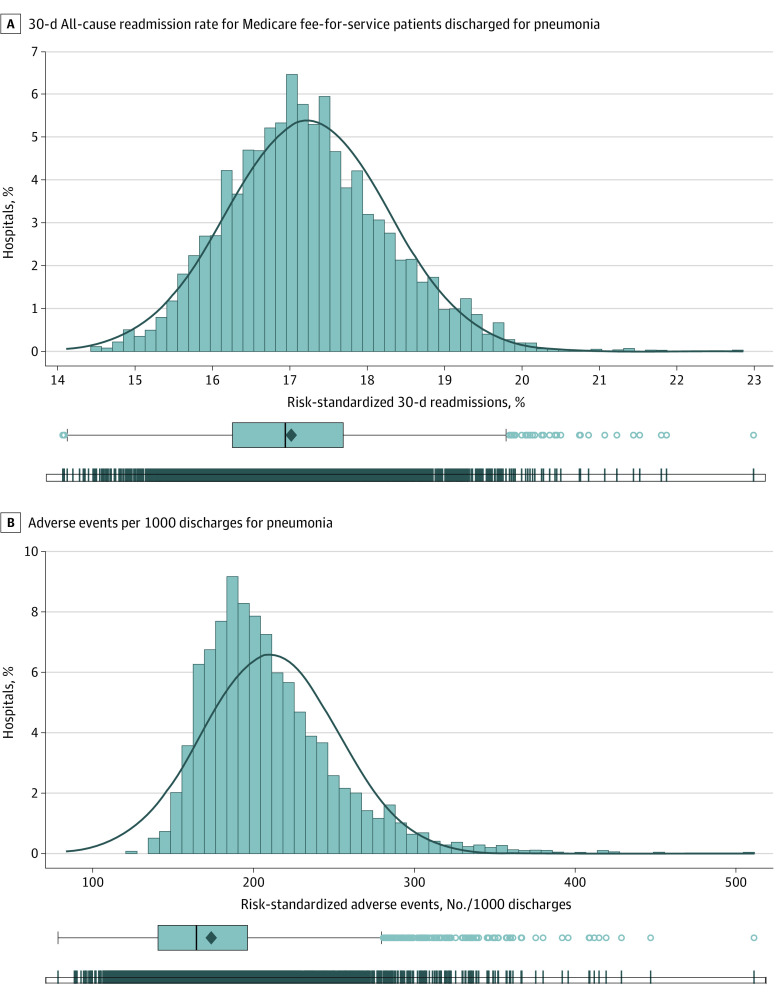
Distribution of Hospital-Specific Risk-Standardized 30-Day All-Cause Readmission Rates and Number of Adverse Events per 1000 Discharges for Pneumonia A, The mean (SD) and median (IQR) of the hospitals’ readmission rate was 17.0% (1.1) and 17.0% (16.3%-17.7%), respectively. One IQR represents a 1.5 percentage point difference between the low and high categories. A total of 2590 hospitals were included. B, The number of adverse events per 1000 discharges was 157.3 (95% CI 152.3-162.5). Line in center of boxes represents the median, with the box boundaries indicating the IQR. Dots indicate individual hospitals.

The linked CMS and MPSMS data included 2590 hospitals and 46 047 patients with pneumonia, with a median (IQR) age of 71 (58-82) years; 23 943 (52.0%) were women, 5305 (11.5%) were Black individuals, 37 763 (82.0%) were White individuals, and 2979 (6.5%) identified as another race (Table). The hospital-specific median (IQR) number of patients was 16 (9-24), and these patients were at risk for 291 895 adverse events; each patient was at risk for a mean (range) of 6.3 (3.0-17.0) adverse events; and the hospital-specific median (IQR) number of adverse events for which patients were at risk was 99 (59-156). Patient characteristics were comparable across hospital performance categories; however, teaching hospitals were more likely to be in the high readmission category (Table). The mean (SD) and median (IQR) of the hospitals’ readmission rate were 17.0% (1.1) and 17.0% (16.3%-17.7%), respectively (Figure 1A). One IQR represents a 1.5 percentage point difference between the low and high categories.

**Table.** Patient and Hospital Characteristics by Hospital Performance on 30-Day Readmissions

Characteristic	Hospital-specific risk-standardized 30-d all-cause readmission rate for patients discharged with pneumonia, No. (%)			
	All	<25th Percentile (range, 14.1% to <16.3%)	25th-75th Percentile (range, 16.3%-17.7%)	>75th Percentile (range, >17.7%-23.0%)
**Patient level**				
Total No.	46 047	11 479	23 403	11 165
Age				
Mean (SD), y	68.8 (16.8)	69.5 (16.6)	68.7 (16.9)	68.4 (16.8)
Age <65 y	16 680 (36.2)	3914 (34.1)	8604 (36.8)	4162 (37.3)
Sex				
Female	23 943 (52.0)	5899 (51.4)	12 209 (52.2)	5835 (52.3)
Male	22 104 (48.0)	5580 (48.6)	11 194 (47.8)	5330 (47.7)
Race				
Black	5305 (11.5)	732 (6.4)	2742 (11.7)	1831 (16.4)
Other^a^	2979 (6.5)	742 (6.5)	1561 (6.7)	676 (6.1)
White	37 763 (82.0)	10 005 (87.2)	19 100 (81.6)	8658 (77.5)
Cancer	10 634 (23.1)	2682 (23.4)	5353 (22.9)	2599 (23.3)
Congestive heart failure	15 412 (33.5)	3812 (33.2)	7793 (33.3)	3807 (34.1)
Chronic obstructive pulmonary disease	21 057 (45.7)	5262 (45.8)	10 607 (45.3)	5188 (46.5)
Cerebrovascular disease	8118 (17.6)	1992 (17.4)	4046 (17.3)	2080 (18.6)
Diabetes	16 348 (35.5)	3946 (34.4)	8290 (35.4)	4112 (36.8)
Obesity	11 432 (24.8)	2951 (25.7)	5776 (24.7)	2705 (24.2)
Smoking	13 478 (29.3)	3373 (29.4)	6817 (29.1)	3288 (29.4)
Coronary artery disease	16 145 (35.1)	3956 (34.5)	8210 (35.1)	3979 (35.6)
Kidney disease	14 951 (32.5)	3799 (33.1)	7446 (31.8)	3706 (33.2)
In-hospital mortality	3294 (7.2)	748 (6.5)	1630 (7.0)	916 (8.2)
Length of stay, mean (SD), d	6 (6.0)	5 (5.3)	6 (5.8)	7 (7.0)
**Hospital level**				
Total No.	2590	647	1296	647
Large teaching	223 (8.6)	31 (4.8)	114 (8.8)	78 (12.1)
Private not-for-profit	1004 (38.8)	273 (42.2)	468 (36.1)	263 (40.6)
Rural hospital	797 (30.8)	254 (39.3)	388 (29.9)	155 (24.0)
JC Accredited	2122 (81.9)	500 (77.3)	1074 (82.9)	548 (84.7)
PCI	1325 (51.2)	351 (54.3)	629 (48.5)	345 (53.3)
CABG	979 (37.8)	255 (39.4)	460 (35.5)	264 (40.8)
No. of hospital beds, mean (SD)	225 (209)	201 (188)	211 (196)	278 (241)

Abbreviations: CABG, coronary artery bypass graft surgery; JC, Joint Commission; PCI, percutaneous coronary intervention.

^a^ Other includes multiracial individuals and those selecting a race other than Black or White.

### Hospital Performance on Readmissions and Patients’ Risk of Adverse Events

**Figure 2.**
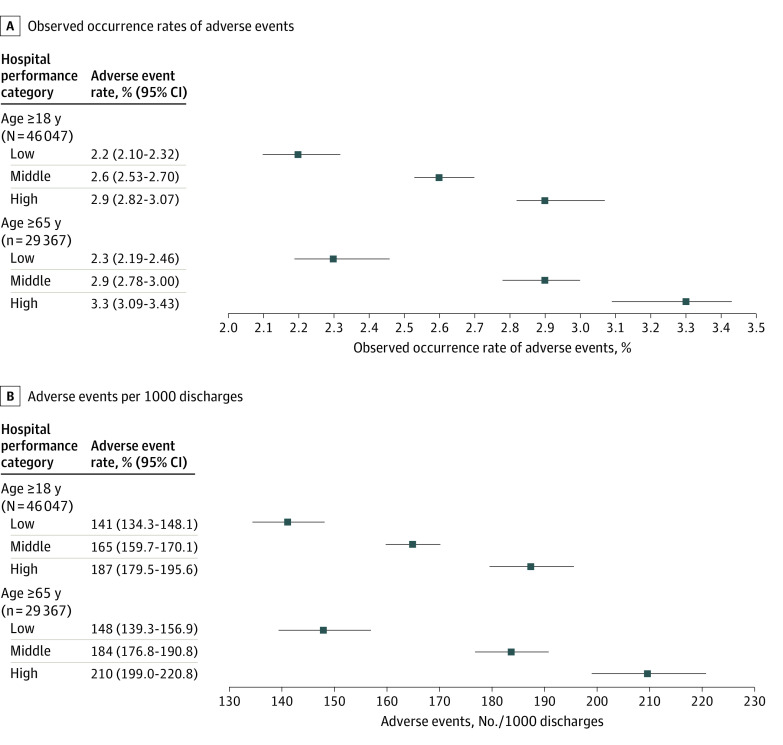
Observed Occurrence Rates of Adverse Events and Adverse Events per 1000 Discharges, by Age Group and Hospital-Specific Risk-Standardized 30-Day All-Cause Admission Rates The hospital-specific risk-standardized 30-day all-cause readmissions category was low if the readmission rate was less than the 25th percentile of the overall rate, high if the readmission rate was greater than the 75th percentile of the overall rate, and average if otherwise. The national occurrence rates of adverse events were 2.6% (95% CI, 2.54%-2.65%) and 2.8% (95% CI, 2.76%-2.91%) for patients aged 18 years and older and 65 years and older, respectively. The national number of adverse events per 1000 discharges were 157.3 (95% CI, 152.3-162.5) and 181.0 (175.7-185.6) for those aged 18 years and older and 65 years and older, respectively.

At the patient level, the overall occurrence rate of adverse events was 2.6% (95% CI, 2.54%-2.65%). Patients who were admitted to hospitals with higher risk-standardized readmission rates were more likely to experience adverse events (Figure 2A and eTable 2 in the Supplement). This finding did not change substantially after accounting for patient and hospital characteristics. An increase by 1 IQR in the risk-standardized readmission rate was associated with a relative 13% increase in the risk of the occurrence rate of adverse events (adjusted odds ratio, 1.13; 95% CI, 1.08-1.17).

**Figure 3.**
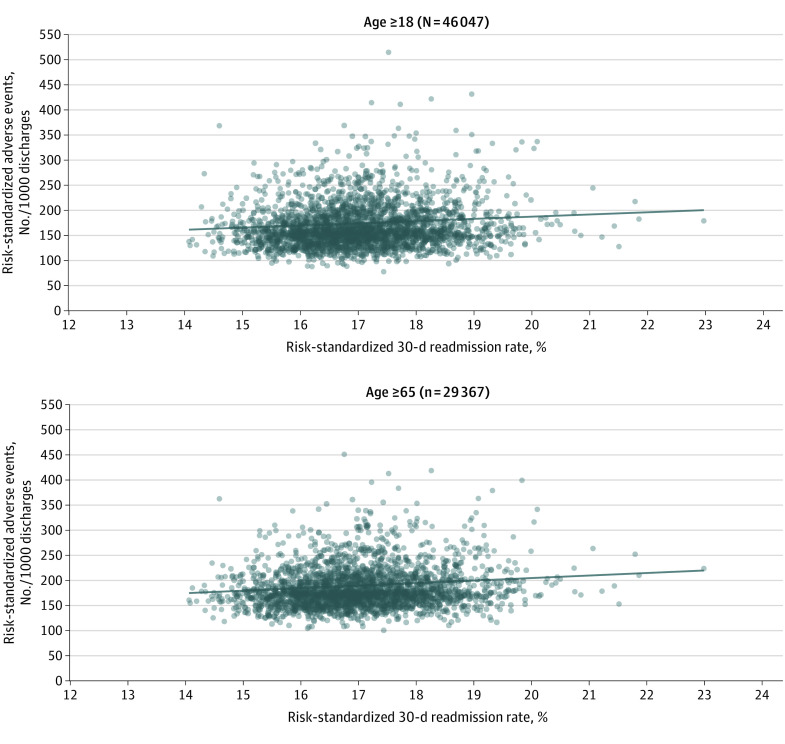
Association Between Hospital-Specific Risk-Standardized 30-Day All-Cause Readmission Rate and Hospital-Specific Risk-Standardized Number of Adverse Events per 1000 Discharges Observed slopes (SE) of regression lines were 4.7 (0.77) for patients aged 18 years and older and 5.0 (0.71) for patients aged 65 years and older. Dots represent individual hospitals.

At the hospital level, the number of adverse events per 1000 discharges was 157.3 (95% CI 152.3-162.5) (Figure 1B). Hospital performance on readmissions was associated with hospital performance on hospital-acquired adverse events (Figure 3). This association persisted after adjusting for hospital characteristics. An increase in 1 IQR in the risk-standardized readmission rate was associated with an increase of 5.0 (95% CI, 2.8-7.2) adverse events per 1000 discharges.

The second analysis, which was restricted to patients aged 65 years or older, included 29 367 patients across 2536 hospitals (eTable 3 in the Supplement) and showed a similar association (Figure 2B and Figure 3). An increase by 1 IQR in the risk-standardized readmission rate was associated with a relative 15% increase in the risk of the occurrence rate of adverse events (adjusted odds ratio, 1.15; 95% CI, 1.10-1.20) and an increase of 6.0 (95% CI, 3.9-8.0) adverse events per 1000 discharges.

The sensitivity analysis, which included hospitals in the first CMS reporting period (July 1, 2010, to June 30, 2013) and the MPSMS data after this reporting period (July 1, 2013, to December 31, 2019), included 15 726 patients across 1429 hospitals. An increase by 1 IQR in the risk-standardized readmission rate was associated with a relative 11% increase in the risk of the occurrence rate of adverse events (adjusted odds ratio, 1.11; 95% CI, 1.04-1.19) and an increase of 6.0 (95% CI, 2.69-8.80) adverse events per 1000 discharges.

## Discussion

In this large and comprehensive investigation of the association between hospital readmission rates and patients’ risk of adverse events during hospitalizations for patients hospitalized with pneumonia, we observed that hospital performance on readmissions was associated with both individual patients’ risk of adverse events and hospitals’ overall performance on adverse events. Our findings are consistent with several possible explanations. The quality domains encompassed by readmission and safety may be similar, and thus the rates track together. It may be that readmission rates are associated with unmeasured patient or hospital factors and those factors are also associated with the risk of adverse events. For example, hospital culture—defined as the set of values and attitudes which govern everyday practice—is increasingly understood to influence patient safety, and such effects may extend both to readmissions and to adverse events. We did adjust for many patient and hospital characteristics but cannot exclude the possibility that the association may have been confounded. Our secondary and sensitivity analyses found that the association between hospital performance on readmissions and patient risk of adverse events was comparable with the main analysis, strengthening the finding that patients admitted to a hospital with high readmission rates may have a higher risk of hospital-acquired adverse events.

Our findings have important implications. The study indicates that readmissions and adverse event rates for patients with pneumonia are associated with each other. The CMS readmission measure for pneumonia could be used as a proxy for the risk of patient safety events. A further implication is that the factors to promote better outcomes in reducing unplanned readmissions may also support higher patient safety performance as indicated by lower adverse events.^18,19^ Hospitals may consider focusing on improving both readmissions and adverse event rates together to result in overall better quality of care. The statistical modeling approach allows us to answer the research question at both patient and hospital levels. Our findings extend previous studies focused on a hypothesis that a high rate of adverse events is associated with a high rate of readmission. Our study assessed the association between hospital performance on readmissions and patients’ risk of developing adverse events and found that the hospital’s risk-standardized 30-day all-cause readmission rate may be used to estimate patients’ risk of hospital-acquired adverse events for patients with pneumonia.

### Limitations

This study has limitations. We focused on adverse events that were both detected and documented during the index hospitalization but were unable to identify events that occurred but were not documented or not included in the MPSMS measures, such as surgical site infections, allergic reactions, and opioid-related adverse events. Variation in the completeness of documentation may affect hospital’s adverse events. Restricted by the MPSMS sample size, we were not able to determine whether hospital performance on readmissions was more strongly associated with some adverse events vs others. Not all adverse events measured by MPSMS data are preventable, but each of these events is thought to be frequently preventable with the delivery of high-quality care.^12,20,21^ The MPSMS data lacks information on patients’ severity of acute illness, which may impact the risk of developing adverse events.

## Conclusions

In this study, patients who were admitted to a hospital with high readmissions were more likely to develop adverse events during their index hospitalization. This finding strengthens the evidence that readmissions reflect quality of hospital care, at least for patients hospitalized with pneumonia.

## Notes

Supplement.
eAppendix 1.

eAppendix 2.
**eTable 1.** List of the 21 Adverse Events for Which Patients Were at Risk During Hospitalizations **eTable 2.** Individual Adverse Events by Hospital Performance on 30-Day Readmissions **eTable 3. **Patient and Hospital Characteristics by Hospital Performance on 30-Day Readmissions Among Patients Aged 65 Years or Older
