# Supplementary material for: Analysis of Hospital-Level Readmission Rates and Variation in Adverse Events Among Patients With Pneumonia in the United States
Source: JAMA Netw Open. 2022 May 31;5(5):e2214586. doi: 10.1001/jamanetworkopen.2022.14586 (PMC9157270; doi:10.1001/jamanetworkopen.2022.14586)
Supplement: Supplement. — eAppendix 1. eAppendix 2. eTable 1. List of the 21 Adverse Events for Which Patients Were at Risk During Hospitalizations eTable 2. Individual Adverse Events by Hospital Performance on 30-Day Readmissions eTable 3. Patient and Hospital Characteristics by Hospital Performance on 30-Day Readmissions Among Patients Aged 65 Years or Older [file jamanetwopen-e2214586-s001.pdf]

Supplemental Online Content

Wang Y, Eldridge N, Metersky ML, et al. Analysis of hospital-level readmission rates and variation in adverse events among patients with pneumonia in the United States. *JAMA Netw Open*. 2022;5(5):e2214586. doi:10.1001/jamanetworkopen.2022.14586

eAppendix 1.
eAppendix 2.
eTable 1. List of the 21 Adverse Events for Which Patients Were at Risk During Hospitalizations
eTable 2. Individual Adverse Events by Hospital Performance on 30-Day Readmissions
eTable 3. Patient and Hospital Characteristics by Hospital Performance on 30-Day Readmissions Among Patients Aged 65 Years or Older

This supplemental material has been provided by the authors to give readers additional information about their work.

© 2022 Wang Y et al. *JAMA Network Open.*

eAppendix 1.

Using a mixed modeling approach that accounts for patient characteristics and permits hospital-level random intercepts, CMS calculates a risk-standardized ratio, defined as hospital-specific "predicted" readmissions divided by hospital-specific "expected" readmissions, for each hospital. The "predicted" readmissions in a hospital were estimated by using the same patient mix with a hospital-specific intercept and the "expected" readmissions in a hospital were estimated by using the same patient mix but with an overall intercept. This ratio, multiplied by the overall national readmission rate, is the risk-standardized readmission rate for an index hospital. A higher than expected readmission rate is indicated when the risk-standardized rate is greater than the national rate.

© 2022 Wang Y et al. *JAMA Network Open.*

eAppendix 2.

To determine whether hospital performance on readmissions is associated with hospital performance on hospital-acquired adverse events, we first fit a mixed model with a Poisson link function and hospital random intercepts to model the number of adverse events as a function of patient characteristics, the time variable, and seasonal indicators. Using this model, we estimated a hospital-specific risk-standardized number of adverse events per 1,000 discharges for each hospital. We then linked it with the CMS readmissions data at the hospital level and fit a mixed model to regress the hospital-specific risk-standardized number of adverse events per 1,000 discharges as a function of the hospital-specific risk-standardized 30-day readmission rate, adjusting for the hospital characteristics described previously. We included State-specific random intercepts in the model to account for within-state and between-state variation and weighted the model by the total number of pneumonia patients in the CMS readmission data.

© 2022 Wang Y et al. *JAMA Network Open.*

eTable 1. List of the 21 Adverse Events for Which Patients Were at Risk During Hospitalizations

Adverse Events Associated with Digoxin
Adverse Events Associated with Hypoglycemic Agents
Adverse Events Associated with Heparin
Adverse Events Associated with Low Molecular Weight Heparin and Factor Xa Inhibitors
Adverse Events Associated with Warfarin
Hospital-Acquired Pressure Ulcers
Inpatient Falls
Central Line-Associated Blood Stream Infections
Postoperative Pneumonia
Hospital-Acquired Antibiotic-Associated Clostridium difficile
Catheter-Associated Urinary Tract Infections
Hospital-Acquired Methicillin-Resistant Staphylococcus aureus
Hospital-Acquired Vancomycin-Resistant Enterococcus
Ventilator-Associated Pneumonia
Adverse Events Associated with Hip Joint Replacement
Adverse Events Associated with Knee Joint Replacement
Mechanical Complications Associated with Central Lines
Postoperative Venous Thromboembolic Events
Postoperative Cardiac Events (Cardiac and Non-cardiac Surgeries)
Adverse Events Associated with Femoral Artery Puncture for Catheter Angiographic Procedures
Contrast Nephropathy Associated with Catheter Angiography

© 2022 Wang Y et al. *JAMA Network Open.*

eTable 2. Individual Adverse Events by Hospital Performance on 30-Day Readmissions

Measure	Hospital-specific risk-standardized 30-day all-cause readmission rate for patients discharged with pneumonia					
	Low (< 25th percentile, range: 14.1% to <16.3%)		Average (25th -75th percentile, range: 16.3%-17.7%)		High (>75th percentile, range: >17.7%-23.0%)	
	Exposure (#)	Rate (95% CI)	Exposure (#)	Rate (95% CI)	Exposure (#)	Rate (95% CI)
Adverse Drug Events Associated with Digoxin	404	0.5 (0.1-1.8)	776	1.0 (0.5-2)	404	0.7 (0.2-2.2)
Adverse Drug Events Associated with Hypoglycemic Agents	4032	9.0 (8.2-10)	8249	10.1 (9.5-10.8)	4100	10.3 (9.4-11.2)
Adverse Drug Events Associated with IV Heparin	300	14.7 (10.9-19.2)	708	18.4 (15.6-21.4)	388	17.3 (13.6-21.4)
Adverse Drug Events-Associated with Low Molecular Weight Heparin and Factor Xa Inhibitor	5211	2.7 (2.3-3.2)	9298	2.9 (2.5-3.2)	4289	4.1 (3.5-4.7)
Adverse Drug Events Associated with Warfarin	1231	5.8 (4.5-7.2)	2211	8.7 (7.5-9.9)	1037	9.5 (7.7-11.4)
Hospital Acquired Pressure Ulcers	11479	5.7 (5.3-6.2)	23403	6.6 (6.3-6.9)	11165	7.3 (6.8-7.8)
In-hospital Patient Falls	11479	0.7 (0.6-0.9)	23403	1.1 (1-1.2)	11165	1.0 (0.8-1.2)
Adverse Drug Events Hospital Acquired Antibiotic Associated Clostridium difficile	11368	0.4 (0.3-0.5)	23137	0.7 (0.6-0.8)	11018	0.9 (0.7-1.1)

© 2022 Wang Y et al. *JAMA Network Open.*

Blood Stream Infection Associated with Central Venous Catheter	106	0.0 (0.0-3.4)	249	1.6 (0.4-4.1)	120	3.3 (0.9-8.3)
Catheter Associated Urinary Tract Infections	2899	2.4 (1.9-3.1)	5700	3.2 (2.8-3.7)	2911	4.4 (3.7-5.2)
Hospital Acquired Methicillin-resistant Staphylococcus Aureus	10955	0.0 (0-0.1)	22240	0.1 (0-0.1)	10512	0.1 (0.1-0.2)
Hospital Acquired Vancomycin Resistant Enterococcus	11416	0.0 (0.0-0.1)	23229	0.0 (0-0.1)	11030	0.1 (0-0.2)
Postoperative Pneumonia	46	41.3 (27-56.8)	69	30.4 (19.9-42.7)	48	14.6 (6.1-27.8)
Ventilator Associated Pneumonia	219	13.7 (9.4-19)	514	13.6 (10.8-16.9)	310	10.6 (7.4-14.6)
Adverse Events Associated with Femoral Artery Puncture for Catheter Angiographic Procedures	65	0.0 (0.0-5.5)	147	2.0 (0.4-5.9)	107	1.9 (0.2-6.6)
Adverse Events Associated with Hip Joint Replacements	3	66.7 (9.4-99.2)	7	14.3 (0.4-57.9)	3	33.3 (0.8-90.6)
Contrast Nephropathy Associated with Catheter Angiography	66	21.2 (12.1-33)	160	20.0 (14.1-27)	98	21.4 (13.8-30.9)

© 2022 Wang Y et al. *JAMA Network Open.*

Mechanical Complications Associated with Central Venous Catheters	1842	3.8 (3-4.8)	3791	3.6 (3-4.2)	2082	4.0 (3.2-4.9)
Postoperative Cardiac/Non-cardiac Arrest Events	79	1.3 (0-6.9)	154	5.2 (2.3-10)	115	7.0 (3.1-13.3)
Postoperative Venous Thromboembolic Event	79	2.5 (0.3-8.9)	154	0.6 (0-3.6)	115	2.6 (0.5-7.4)

© 2022 Wang Y et al. *JAMA Network Open.*

eTable 3. Patient and Hospital Characteristics by Hospital Performance on 30-Day Readmissions Among Patients Aged 65 Years or Older

Characteristics	Hospital-specific risk-standardized 30-day readmission rate for patients hospitalized for pneumonia				
	**Aggregated**	**Low (< 25th percentile, range: 14.1%-16.3%)**	**Average (25th -75th percentile, range: 16.3%-17.7%)**	**High (>75th percentile, range: 17.7%-23.0%)**	
**Patient Level**					
Total (#)	29367	7565	14799	7003	
Age, Mean (SD)	79.2 (8.6)	79.2 (8.6)	79.2 (8.5)	79.0 (8.5)	
Female, # (%)	15405 (52.5)	3925 (51.9)	7744 (52.3)	3736 (53.3)	
Male, # (%)	13962 (47.5)	3640 (48.1)	7055 (47.7)	3267 (46.7)	
Black, # (%)	2358 (8.0)	303 (4.0)	1221 (8.3)	834 (11.9)	
Other, # (%)	1755 (6.0)	444 (5.9)	888 (6.0)	423 (6.0)	
White, # (%)	25254 (86.0)	6818 (90.1)	12690 (85.7)	5746 (82.1)	
Cancer, # (%)	8165 (27.8)	2143 (28.3)	4058 (27.4)	1964 (28.0)	
Congestive Heart Failure, # (%)	11962 (40.7)	3010 (39.8)	6035 (40.8)	2917 (41.7)	
Chronic Obstructive Pulmonary Disease, # (%)	14607 (49.7)	3743 (49.5)	7322 (49.5)	3542 (50.6)	
Cerebrovascular Disease, # (%)	6476 (22.1)	1617 (21.4)	3231 (21.8)	1628 (23.2)	
Diabetes Mellitus, # (%)	10906 (37.1)	2663 (35.2)	5501 (37.2)	2742 (39.2)	
Obesity, # (%)	5803 (19.8)	1542 (20.4)	2894 (19.6)	1367 (19.5)	
Smoking, # (%)	5720 (19.5)	1487 (19.7)	2861 (19.3)	1372 (19.6)	
Coronary Artery Disease, # (%)	12767 (43.5)	3187 (42.1)	6496 (43.9)	3084 (44.0)	
Renal Disease, # (%)	10923 (37.2)	2854 (37.7)	5414 (36.6)	2655 (37.9)	
In-hospital mortality, # (%)	2518 (8.6)	570 (7.5)	1238 (8.4)	710 (10.1)	
Length of stay, mean (SD)	6 (5.3)	5 (4.5)	6 (5.2)	7 (6.4)	
**Hospital Level**					
Total (#)	2536	632	1273	631	

© 2022 Wang Y et al. *JAMA Network Open.*

Large Teaching, # (%)	218 (8.6)	31 (4.9)	111 (8.7)	76 (12.0)	
Private Not-for-profit, # (%)	982 (38.7)	266 (42.1)	461 (36.2)	255 (40.4)	
Rural Hospital, # (%)	789 (31.1)	252 (39.9)	385 (30.2)	152 (24.1)	
JC Accredited, # (%)	2083 (82.1)	490 (77.5)	1056 (83.0)	537 (85.1)	
PCI, # (%)	1296 (51.1)	343 (54.3)	618 (48.5)	335 (53.1)	
CABG, # (%)	959 (37.8)	248 (39.2)	451 (35.4)	260 (41.2)	
Hospital Bed Size, mean (SD)	225 (209)	202 (189)	210 (195)	278 (243)	

SD: Standard deviation; JC: Joint Commission; PCI: percutaneous coronary intervention; CABG: coronary artery bypass graft surgery; Other: any identified race not included in the aforementioned categories and mixed race.

© 2022 Wang Y et al. *JAMA Network Open.*
